# Association of *GALC*, *ZNF184*, *IL1R2* and *ELOVL7* With Parkinson’s Disease in Southern Chinese

**DOI:** 10.3389/fnagi.2018.00402

**Published:** 2018-12-13

**Authors:** Gen Li, Shishuang Cui, Juanjuan Du, Jin Liu, Pingchen Zhang, Yang Fu, Yixi He, Haiyan Zhou, Jianfang Ma, Shengdi Chen

**Affiliations:** ^1^Department of Neurology & Collaborative Innovation Center for Brain Science, Ruijin Hospital, Shanghai Jiao Tong University School of Medicine, Shanghai, China; ^2^Co-innovation Center of Neuroregeneration, Nantong University, Nantong, China

**Keywords:** Parkinson’s disease, single nucleotide polymorphism, *GALC*, *ZNF184*, *IL1R2*, *ELOVL7*

## Abstract

**Study Objectives:** The aim of the study was to investigate the relationship between 22 single nucleotide polymorphisms (SNPs) and Parkinson’s disease (PD) in the Chinese population.

**Methods:** A total of 250 PD patients and 240 healthy controls were recruited. The SNaPshot technique and the polymer chain reaction were used to detect 22 SNPs.

**Results:** rs8005172 of *GALC*, rs9468199 of *ZNF184* and rs34043159 of *IL1R2*, were associated with PD (rs8005172: *p* = 0.009, OR = 0.69, allele model, *p* = 0.010, additive model, *p* = 0.015, OR = 2.17, dominant model; *p* = 0.020, OR = 2.11, dominant model after adjustment; *p* = 0.036, OR = 1.47, recessive model after adjustment; rs9468199: *p* = 0.008, OR = 1.52, allele model, *p* = 0.008, additive model, *p* = 0.007, OR = 0.22, recessive model, *p* = 0.005, OR = 0.20, recessive model after adjustment; rs34043159: *p* = 0.034, OR = 1.31, allele model, *p* = 0.036, additive model).

**Conclusion:** Our study revealed that *GALC*, *ZNF184*, and *IL1R2* were associated with PD in the southern Chinese population. *GALC* was also associated with LOPD. *ELOVL7* and *ZNF184* were associated with EOPD. In addition, trends of association to PD, between *SATB1*, *NMD3*, and *FGF20*, were also found.

**Statement of Significance:** Genetic play an important role in the pathogenesis factors of Parkinson’s disease (PD). We found that *GALC*, *ZNF184*, and *IL1R2* were associated with PD. *GALC* was also associated with late onset of PD, while *ELOVL7* and *ZNF184* were associated with early onset PD. This study is the first to find an association between *GALC*, *ZNF184*, and rs2280104 with PD.

## HIGHLIGHTS

- *GALC*, *ZNF184*, and *IL1R2* were associated with PD.
- *GALC* was also associated with late onset PD.
- *ELOVL7* and *ZNF184* were associated with early onset PD.

## Introduction

Parkinson’s disease is a debilitating and progressive movement disorder characterized by bradykinesia, resting tremors and rigidity. As the second most common neurodegenerative disorder after Alzheimer’s disease, it causes a high burden on the health-care system (Schrag and Schott, 2006; Ascherio and Schwarzschild, 2012; Olesen et al., 2012). The pathological characteristics of PD include an abnormal α-synuclein aggregation and a loss of dopaminergic neurons in the substantia nigra. In addition, several hypotheses of the pathogenesis of PD, such as autophagy and mitochondrial dysfunction, have been raised previously (Dehay et al., 2013; Wang and Mao, 2014; Wang et al., 2016; Xilouri et al., 2016).

Genetic factors play an important role in the pathogenesis of PD. With the help of GWAS, several PD-associated locus, such as *LRRK2*, were discovered (Trinh and Farrer, 2013). Recently, using a meta-analysis of GWAS, several PD risk loci such as *GALC* and *IL1R2*, etc., have been identified, mainly in Caucasians (Nalls et al., 2014; Chang et al., 2017). However, because of different allele frequencies across ethnicities, we cannot infer distributions of these loci in Asians. Additionally, the association between these loci and PD in the southern Chinese population, was unclear. In this study, we attempted to replicate these loci demonstrated in these two previous articles (Nalls et al., 2014; Chang et al., 2017) and discussed those genes associated with PD in the southern Chinese population.

## Materials and Methods

### Study Population

Parkinson’s disease was diagnosed by movement disorder specialists, based on diagnostic criteria provided by the MDS (Postuma et al., 2015). Control subjects from a healthy community were enrolled and evaluated by movement disorder specialists for exclusion of PD. Hoehn – Yahr staging and familial history were recorded for patients with PD. When assessing PD patients, secondary causes, such as inflammatory, drug-induced, vascular and toxin-induced parkinsonism were all excluded. Parkinsonism along with other neurodegenerative diseases, such as progressive supranuclear palsy, multiple system atrophy and Wilson’s disease were also excluded. We divided the PD patients into a late onset PD group (LOPD) and an early onset PD group (EOPD). Patients who were above 45 years old when PD was first diagnosed, were regarded as LOPD (Gonzalez-Del Rincon Mde et al., 2013). Mild PD was defined when Hoehn – Yahr staging was below 2.5 after assessment. Patients with relatives who have PD (within the last three generations), were regarded as familial PD patients. All participants signed consent forms and this study was approved by the ethics committee of the Ruijin Hospital affiliated to Shanghai Jiao Tong University School of Medicine.

### DNA Preparations and Genotyping

Two milliliter blood samples were collected from PD patients and healthy controls. A phenol-chloroform-isopropyl alcohol method was used to extract the DNA. The Primer Premier 5 (version 5.00, PREMIER Biosoft International) was used to design the primers. The following SNPs were tested: rs4784227 of *TOX3*, rs2280104 of *SORBS3/PDLIM2/C8orf58/BIN3*, rs11868035 of *SREBF/RAI1*, rs11343 of *COQ7*, rs591323 of *FGF20*, rs7077361 of *ITGA8*, rs8005172 of *GALC*, rs34016896 of *NMD3*, rs2694528 of *ELOVL7*, rs10906923 of *FAM171A1*, rs9468199 of *ZNF184*, rs60298754 of *MMP16*, rs4073221 of *SATB1*, rs143918452 of *ALAS1/TLR9/DNAH1/BAP1/PHF7/NISCH/STAB1/ITIH3/ITIH4*, rs12497850 of *NCKIPSD/CDC71*, rs4653767 of *ITPKB*, rs34043159 of *IL1R2*, rs601999 of *ATP6V0A1/PSMC3IP/TUBG2*, rs2823357 of *USP25*, rs2740594 of *CTSB* and rs78738012 of *ANK2/CAMK2D*. The SNaPshot technique was used to genotype SNPs. The polymer chain reaction was used to detect the rs13294100 of *SH3GL2.* Details of primers and the reaction system of the polymer chain reaction are described in Supplementary Table S1.

### Statistical Analysis

R (version 3.5.0) was used to perform the statistical analysis. The CATT package (version 2.0), epitools package (version 0.5–10) and the survival package (version 2.41–3) were used in R. A *t-*test was used to compare the differences in age between PD patients and controls. A chi-square test was used to compare the differences in gender, assessing the HWE and the allele distribution between PD patients and controls. Risk analysis was performed by a logistic regression model, and an OR, with a 95% confidence interval for each SNP, according to the dominant, recessive and overdominant models, was calculated. Age and gender were adjusted accordingly. The Cochran-Armitage trend test was used to calculate additive models. We corrected the *p*-values using the Bonferroni method. An AFT model was used to assess the relationship between SNPs and PD over time. We assumed the distribution as a logistic in the AFT model. The logLik results were used to model the evaluation. The genetic power was calculated using Power and Sample Size Calculations (version 3.1.2) (Dupont and Plummer, 1990, 1998).

## Results

There was no difference between age and gender in PD patients and control groups. The PD group was predominantly male (58.0%), and the average age was 54.69 ± 13.95 years. 72.0% of the PD patients had mild PD, and 10.80% PD patients had familial PD. 33 (13.20%) of the PD patients had EOPD. In the EOPD patients, 14 (42.42%) were female and 28 (84.84%) of them had mild PD, after assessment, while 4 (12.12%) of them had familial PD. In the LOPD patients, 91 (41.94%) were female, and 152 (69.58%) of them had mild PD, while 23 (10.60%) of them had familial PD. There were no discrepancies in gender between EOPD and LOPD patients, mild PD or familial PD patients (gender: *p* = 1.000, chi-square test; mild PD: *p* = 0.1196, chi-square test; familial PD: *p* = 0.765, fisher test) (Table 1).

**Table 1:** Demographic data of cases and controls.

	PD patients (*N* = 250)	Controls (*N* = 240)	*p*-value
Gender, female, N (%)	105 (42.00)	100 (41.67)	0.940
Age, mean (SD), years	54.69 (13.95)	56.26 (7.25)	0.344
Mild PD, N (%)^a^	180 (72.00)	–	–
Familial PD, N (%)	27 (10.80)	–	–
EOPD, N (%)	33 (13.20)	–	–
Gender, female, N (%)^b^	14 (42.42)	–	–
Mild PD, N (%)^b^	28 (84.84)	–	–
Familial PD, N (%)^b^	4 (12.12)	–	–
LOPD, N (%)	217 (86.80)	–	–
Gender, female, N (%)^c^	91 (41.94)	–	–
Mild PD, N (%)^c^	152 (69.58)	–	–
Familial PD, N (%)^c^	23 (10.60)	–	–


*EOPD, early onset Parkinson’s Disease; LOPD, late onset Parkinson’s Disease; PD Parkinson’s disease; SD, standard deviations. ^*a*^Assessed by Hoehn – Yahr Staging. ^*b*^Referred to EOPD. ^*c*^Referred to LOPD.*

All SNPs were in the HWE except for the rs13294100 of *SH3GL2*. The rs2740594 of *CTSB* were all an AA genotype, while the rs7077361 of *ITGA8* and rs78738012 of *ANK2/CAMK2D* were all a TT genotype. The rs8005172 of *GALC*, rs9468199 of *ZNF184* and the rs34043159 of *IL1R2* were all associated with PD, in both the allele model as well as the additive model (rs8005172: *p* = 0.009, OR = 0.69, allele model, *p* = 0.010, additive model; rs9468199: *p* = 0.008, OR = 1.52, allele model, *p* = 0.008, additive model; rs34043159: *p* = 0.034, OR = 1.31, allele model, *p* = 0.036, additive model). The dominant model of the rs8005172 of *GALC*, was associated with PD, with or without adjusting the age and gender (*p* = 0.015, OR = 2.17; *p* = 0.020, OR = 2.11, after adjustment). Under recessive models, the rs9468199 of *ZNF184* as well as the rs8005172 of *GALC*, were associated with PD after adjustment (rs9468199: *p* = 0.007, OR = 0.22, *p* = 0.005, OR = 0.20, after adjustment; rs8005172: *p* = 0.036, OR = 1.47, after adjustment). None were statistically significant after the Bonferroni correction (Table 2 and Supplementary Table S2).

**Table 2:** Association of SNPs of candidate genes and odds ratio to PD risk.

	Candidate Gene	SNP	Model	*p*-value^a^	OR^a^	95% CI^a^
PD vs. control	*GALC*	Rs8005172	Allele model	0.009	0.69	(0.53, 0.92)
	*GALC*	Rs8005172	Additive model^c^	0.010	–	–
	*ZNF184*	rs9468199	Allele model	0.008	1.52	(1.12, 2.08)
	*ZNF184*	rs9468199	Additive model^c^	0.008	–	–
	*IL1R2*	rs34043159	Allele model	0.034	1.31	(1.02, 1.69)
	*IL1R2*	rs34043159	Additive model^c^	0.036	–	–
	*GALC*	rs8005172	Dominant model	0.015	2.17	(1.18, 4.15)
	*GALC*	rs8005172	Dominant model^b^	0.020	2.11	(1.14, 4.07)
	*GALC*	rs8005172	Recessive model^b^	0.036	1.47	(1.03, 2.12)
	*ZNF184*	rs9468199	Recessive model	0.007	0.22	(0.06, 0.60)
	*ZNF184*	rs9468199	Recessive model^b^	0.005	0.20	(0.06, 0.56)
LOPD vs. control	*GALC*	rs8005172	Allele model	0.016	0.71	(0.53, 0.94)
	*GALC*	rs8005172	Additive model^c^	0.016	–	–
	*GALC*	rs8005172	Dominant model	0.014	2.22	(1.19, 4.29)
	*GALC*	rs8005172	Dominant model^b^	0.011	2.39	(1.23, 4.81)
	*GALC*	rs8005172	Recessive model^b^	0.046	1.48	(1.01, 2.18)
EOPD vs. control	*ELOVL7*	rs2694528	Allele model	0.032	2.24	(1.05, 4.77)
	*ELOVL7*	rs2694528	Additive model^c^	0.029	–	–
	*ZNF184*	rs9468199	Allele model	0.0001	2.82	(1.62, 4.88)
	*ZNF184*	rs9468199	Additive model^c^	0.0001	–	–
	*ELOVL7*	rs2694528	Dominant model	0.024	2.60	(1.10, 5.82)
	*ZNF184*	rs9468199	Dominant model	0.024	2.34	(1.12, 4.95)
	*ZNF184*	rs9468199	Recessive model	<0.0001	0.06	(0.02, 0.23)
	*ZNF184*	rs9468199	Recessive model^b^	0.014	0.12	(0.02, 0.62)
	*ELOVL7*	rs2694528	Overdominant model	0.019	0.37	(0.17, 0.88)


*CI: Confidence Interval, EOPD: early onset Parkinson’s disease, LOPD: late onset Parkinson’s disease, OR: odd ratio, PD: Parkinson’s Disease; SNP: single nucleotide polymorphism^.^^*a*^*p*-value, OR and 95% CI were obtained from risk analysis and refer to the risk allele. ^*b*^Adjusted for age and gender. ^*c*^*p*-value was calculated by Cochran-Armitage trend test for additive model.*

As for LOPD, all SNPs were in the HWE except for the rs13294100 of *SH3GL2*. The rs8005172 of *GALC* was associated with PD in both the allele model as well as the additive model (rs8005172: *p* = 0.016, OR = 0.71, allele model, *p* = 0.016, additive model). The dominant model of the rs8005172 of *GALC*, with or without adjustment as well as the recessive model of the rs8005172 of *GALC*, after adjustment, were both associated with PD (dominant model: *p* = 0.014, OR = 2.22; *p* = 0.011, OR = 2.39, after adjustment; recessive model: *p* = 0.046, OR = 1.48, after adjustment). None were statistically significant after the Bonferroni correction (Table 2 and Supplementary Table S3).

In the analysis between EOPD and PD, all SNPs were in the HWE except for the rs13294100 of *SH3GL2*. The genetic power of the rs4784227 of *TOX3*, was insufficient. In the remaining SNPs, the rs2694528 of *ELOVL7* and the rs9468199 of *ZNF184* were associated with PD, in both the allele and additive model (rs2694528: *p* = 0.032, OR = 2.24, allele model, *p* = 0.029, additive model; rs9468199: *p* = 0.0001, OR = 2.82, allele model, *p* = 0.0001, additive model). The dominant models of the rs2694528 of *ELOVL7* and the rs9468199 of *ZNF184*, were associated with PD before adjustment (rs2694528: *p* = 0.024, OR = 2.60; rs9468199: *p* = 0.024, OR = 2.34). The recessive model of the rs9468199 of *ZNF184*, was associated with PD with or without adjustment (*p* < 0.0001, OR = 0.06; *p* = 0.014, OR = 0.12, after adjustment). The overdominant model of the rs2694528 of *ELOVL7*, was associated with PD (*p* = 0.019, OR = 0.37). After correcting the *p*-values, the allele model, additive model and the recessive model of the rs9468199 of *ZNF184*, remained statistically significant (Table 2 and Supplementary Table S4).

Within these models, we found that the combination of the dominant model of the rs8005172 of *GALC* and the recessive model of the rs9468199 of *ZNF184*, could better interpret the risk of PD. In LOPD, the dominant model of the rs8005172 of *GALC*, was better. As for EOPD, the combination of the overdominant model of the rs2694528 of *ELOVL7* and the rs9468199 of *ZNF184*, could over time, interpret the risk better (Table 3 and Supplementary Table S5).

**Table 3:** Accelerated failure time models of genetic models of SNPs of each comparison.

	Models of SNPs	*p*-value	Coefficients value	95% CI
PD vs. control	Dominant model of rs8005172	3.40 × 10^-2^	-3.49	(-6.72, -0.26)
	Recessive model of rs9468199	1.18 × 10^-3^	8.23	(3.25, 13.20)
LOPD vs. control	Dominant model of rs8005172	2.85 × 10^-2^	-2.98	(-5.65, -0.31)
EOPD vs. control	Overdominant model of rs2694528	5.04 × 10^-3^	18.89	(5.69, 32.09)
	Recessive model of rs9468199	4.84 × 10^-6^	41.61	(23.77, 59.45)


*AFT: Accelerated failure time; CI: confidence interval; EOPD: Early Onset Parkinson’s Disease; LOPD: Late Onset Parkinson’s Disease; PD: Parkinson’s Disease; SNP: single nucleotide polymorphisms.*

## Discussion

In our study, we found that the following SNPs were associated with PD: the rs8005172 of *GALC* (allele model, additive model, dominant model, recessive model after adjustment), the rs9468199 of *ZNF184* (allele model, additive model, recessive model) and the rs34043159 of *IL1R2* (allele model, additive model). The rs8005172 of *GALC* (allele model, additive model, dominant model, recessive model after adjustment) was associated with LOPD, while the rs2694528 of *ELOVL7* (allele model, additive model, dominant model, overdominant model) and the rs9468199 of *ZNF184* (allele model, additive model, dominant model, recessive model) were associated with EOPD. We also found that the rs4073221 of *SATB1*, the rs34016896 of *NMD3* and the rs591323 of *FGF20* were all associated with PD in the southern Chinese in trend. This research, is the first to replicate the associations of *GALC*, *ZNF184*, *ELOVL7*, and *IL1R2* with PD, in the southern Chinese population. We failed to replicate the association of the remaining loci, identified in Caucasians, with PD in the Chinese population. As ethnicities differ (between Caucasians and Chinese) and because of very few interracial marriages, allele frequencies and the genetic biomarkers of PD might also differ.

*GALC*, which encodes galactocerebrosidase, is the cause of Krabbe disease, a lysosomal storage disorder (Marshall and Bongarzone, 2016). α-synuclein can be degraded and eliminated by autophagy, which could maintain the homeostasis of the dopaminergic neurons (Mishra et al., 2015). Dysfunction of *GALC* could increase autophagy. In *GALC* deficient cells, autophagy markers were increased (Ribbens et al., 2014). The deposition of α-synuclein can also be observed in Krabbe disease (Smith et al., 2014). Synaptic function, influenced by psychosine, may be associated with the pathogenesis of PD (Marshall and Bongarzone, 2016). Similarly, Gaucher disease, a lysosomal storage disorder in which α-synuclein was also found, is associated with PD via several links such as genetics, autophagy impairment, and mitochondrial dysfunction (DePaolo et al., 2009; Velayati et al., 2010; Shachar et al., 2011). Our findings provide more proof of the association between lysosomal storage disorder and PD, and support the theory of autophagy in the pathogenesis of PD.

Protein encoded by *ZNF184* belong to the Kruppel-like zinc finger family and may be involved in transcriptional regulation. The function of *ZNF184* is still unknown. As previously reported, *ZNF184* was associated with aberrant cell proliferations, such as lung cancer and choriocarcinoma (Li et al., 2005; ?). A *trans*-CpG nearest to ZNF184, associated with genetically defined elevated homocysteine (*MTHFR* C677T), was observed (Mandaviya et al., 2017). Hyperhomocysteinemia is a treatable risk factor of PD. *MTHFR* C677T was associated with the increased risk of PD (Wu et al., 2013). Higher serum homocysteine could predict higher dopaminergic neurodegeneration in the substantia nigra (Haghdoost-Yazdi et al., 2014). In addition, hyperhomocysteinemia was also associated with the deterioration of several cognitive functions (memory, verbal fluency, etc.) in PD (Licking et al., 2017). The relationship between *ZNF184*, hyperhomocysteinemia and PD still needs to be investigated further.

The product of *ELOVL7* is a member of the elongase family, ELOVLs, catalyzing the elongation of very long-chain fatty acids. The ELOVL7 protein, presented high activity toward acyl-CoAs with a C18 carbon chain length (Naganuma et al., 2011). It is an important lipogenic enzyme that is involved in the pathogenesis of prostate cancer(Tamura et al., 2009). According to the function of ELOVLs, there is a chance that*ELOVL7* could be involved in the pathogenesis of oxidative stress. Recent studies also indicated that *ELOVL7*, with the help of GWAS, was associated with multiple system atrophy (Sailer et al., 2016; Gu et al., 2018). However, the link between *ELOVL7* and neurodegeneration is still unknown. In this study, we found that *ELOVL7* is associated with EOPD but not with PD. It might indicate that *ELOVL7* plays a more important role at the onset of EOPD, rather than in PD. Furthermore, the population that we tested was small. Larger studies are needed to test this association.

*IL1R2* expresses the receptor of IL1. The association between IL1 and dopaminergic dysfunction has been proven. In the serum of PD patients, the level of IL1-β was higher than in the control patients (Williams-Gray et al., 2016). The *IL1B* gene was associated with restless legs syndrome (Hennessy et al., 2014). The increased levels of IL1, in patients suffering from depression, were also observed (Maes et al., 2012). It may indicate that immunological factors are involved in the pathogenesis of PD.

There were some limitations in our study. First, the genetic power of most SNPs in our study was low. As a single centered study, it was difficult to obtain a large population. Similar studies, in the Asian population, are needed to confirm our findings. Second, we did not create subgroups, to analyze differences between other confounders such as gender, PD subtypes, severity of PD, etc., Because of the small sample, validity might be lost, therefore, larger sized cohort studies are warranted.

Our study revealed that *GALC*, *ZNF184*, and *IL1R2* are associated with PD in the southern Chinese population. *GALC* is also associated with LOPD, while *ELOVL7* and *ZNF184* are associated with EOPD. Additionally, trends of the association of *SATB1*, *NMD3*, and *FGF20* with PD, were also found. Since these genes were identified in both Asian and Caucasian groups, research on the pathogenesis of these genes to PD is needed.

## Ethics Statement

This study was carried out in accordance with the recommendations of the ethic committee of Ruijin Hospital affiliated to Shanghai Jiao Tong University School of Medicine with written informed consent from all subjects. All subjects gave written informed consent in accordance with the Declaration of Helsinki. The protocol was approved by the ethic committee of Ruijin Hospital affiliated to Shanghai Jiao Tong University School of Medicine.

## Author Contributions

GL and SC collected the PD and control patient data, performed the statistical analysis and drafted the manuscript. JL, JD, PZ, YF, and YH collected the data on PD. SC, JM, and HZ designed the study, supervised the study, double-checked the statistical analysis and revised the manuscript.

## Conflict of Interest Statement

The authors declare that the research was conducted in the absence of any commercial or financial relationships that could be construed as a potential conflict of interest.

## Abbreviations

AFT: accelerated failure time
EOPD: early onset Parkinson’s disease
GWAS: genome-wide association studies
HWE: Hardy-Weinberg equilibrium
IL1: interleukin 1
LOPD: late onset Parkinson’s disease
MDS: movement disorders society
OR: odds ratio
PD: Parkinson’s disease
SNP: single nucleotide polymorphism
