# Supplementary material for: Association of GALC, ZNF184, IL1R2 and ELOVL7 With Parkinson’s Disease in Southern Chinese
Source: Front Aging Neurosci. 2018 Dec 13;10:402. doi: 10.3389/fnagi.2018.00402 (PMC6301214; doi:10.3389/fnagi.2018.00402)
Supplement: Supplementary file 1 [file Table_1.DOCX]

Table S1 Primers of tested single nucleotide polymorphisms

| SNPs | Forward Primers | Reverse Primers | Elongation Primers |
| --- | --- | --- | --- |
| rs34043159 | AAA GGA TAG ATG GAT CTT GAC CAC ACT | TGC CCA ATA TCT TCC CAA CCC | TCT GCT CTG ACA AAT CTG CTA AA |
| rs4653767 | ATG CCC AAG TAA AGC TCA ACA GA | TTG TCT GAT ACG GAA TGA CTT GTT AC | AGA TCA GAT TTC TAA CAG TGA ATT C |
| rs2823357 | GTT ATA ACT GAT CTA TAT GCT TTG TGA AG | GCA CTA TAC CAA GAA ATG TGG CAA TT | CAG AGT CTA ACA TTC ATT AAG CAT CA |
| rs601999 | CCA GGC ACA TGG ATG AGT TCT T | CTC ACA TGA CCA TGG TTA CAG TCT T | CAA GGC CAG TCC AGA TTC TT |
| rs2694528 | GAG AAC CAG AAG CAG AGA TGA GTG AT | GTG ACA GGG CAC TGA GTT CTA ACG | TTG GGG AAG GGA GAG TGC |
| rs143918452 | TGC CTT CTC CAC CTC CCT CAT | CAC CTT CCA ACT CCA AAT ACC CAT | GAG ATA GGA CAA CCA GGT GGC |
| rs12497850 | CAG AGG CAG CCC AAA GAC C | GTT GCT GAG ATT ATG ATC TTC CTA AAT | AGT AGT GGG TTT GTA ATC AAC TGG |
| rs9468199 | TCA TCT CCA TAA ATC CTA CCC TTA CTG | AGA CGC TTC TGG AGG GAA GAG | GCA GGG GTA TTT TGT TGT CC |
| rs10906923 | TCA GTT TCT TCT GTT GCA CTG TAA CG | CGT TTG CTG CTT GCA TAG TAT CTG T | GAC ATG ACA AGC ATC ACA GGA |
| rs591323 | CAT GTC CTT TGC AGR AAC GC | CCA CCC TCA ACC CTC CGA AT | TGG GAG CTA AAT GGT GAT AAC AC |
| rs4073221 | TGT GTT TAA ATT AAC CAC TGC ATA GTC | TCC TCC TCC TGC TAT CAA ATG C | CAA GGT TAC TTT GAG GAT TAA ATA ACA |
| rs78738012 | TGT CCA TTA ACG GAT GAG TGA ATA C | CCA ATC CCA CCC CAG TCC TAA | AGC AAG CAA CCA CCA CTA ATC T |
| rs11343 | AAA AGA TAC AGA AGC ATC CAA CAG AG | GAT TAA AAT GGC TAT CAC ACC CCA | AGA CGT GAC ATT CGT TCA CAG A |
| rs8005172 | TCT GCT ATG CTG TGG CTA GAT GAC TA | CCA TTG GTG TTT CTT CAC CTC TCT A | AAG CAA AGT TCC TAG CRC ATG |
| rs7077361 | CAT CGG AAG TTG ATC TGA AAG GAT C | AAA GCC ACC CAC CCA CCA | ATG TTC GAG TGC TAC CTC TCA A |
| rs11868035 | GTG AGA CGT GCC AGA CTT CTT G | GGG TCA GTT GTC CCT TCT CAC | TCC CAG CTC TGT CCC CAT |
| rs60298754 | GGG TCC AAG GAT CTT ATA GCT GTG | CAG TTA ACC CGA TGA CAT AAC CAT AT | GAA AAG ATA GCT AAT GGT CTA ACC TAG |
| rs2280104 | CAC TCC GCA CAC ACC CTT CC | GAC TCC CTG GCT TCT TTT CTC AT | GGG CAG GCA GTG AGT AAT AGC |
| rs4784227 | ACA GCC AAC TCT TTG GGG AGG | TGC CTG TTC ATC TCC TTC CAA | AAG TCC CAA TTT GTA GTG TTT GC |
| rs2740594 | AGC TTT TAA AGC AAG AGA AGC ACC | AGG CAC CTA TAA TCC CAG CTA CTA TA | AAT CTC AGC TCA CTG CAAC CT |
| rs34016896 | TTT CAT TTG TCC TGA CAA TCA TGT AG | CCA AGT GGG AGC TAC AAG TTA GTC C | CAT GTA GCT AAA AAT ATG TAT CTA ATT AAG |
| rs13294100 | CAT CAT CTC GCG CCC GT | ACG ACC GAG TTC TCC AGG TA |  |
| SNP: single nucleotide polymorphism | | | |

The reaction system of rs13294100 is following: R Taq (R001A, Takara) 0.25μL, 10× loading buffer (R001A, Takara) 5μL, dNTP Mixture (R001A, Takara) 4μL, ddH2O 37.75μL, Template 1μL, Forward Primer (10 μmol/L) 1μL, Reverse Primer (10 μmol/L) 1μL

Table S2 Association of SNPs of candidate genes and odds ratio to PD risk

| Candidate Gene | SNP | HWE *p* value | MAF (case/control) | Allele | | | | | | | | | Dominant Model | | | |
| --- | --- | --- | --- | --- | --- | --- | --- | --- | --- | --- | --- | --- | --- | --- | --- | --- |
|  |  |  |  | Effect allele | | | | *p^a^* | *p*_trend_^c^ | OR^a^ | 95%CI^a^ | | *p* | OR | | 95%CI |
| *TOX3* | rs4784227 | 0.958 | 0.24/0.23 | T | | | | 0.946 | 0.731 | 1.01 | (0.75, 1.36) | | 0.824 | 0.96 | | (0.67, 1.38) |
| *SORBS3, PDLIM2, C8orf58, BIN3* | rs2280104 | 0.998 | 0.20/0.16 | T | | | | 0.116 | 0.120 | 1.30 | (0.94, 1.82) | | 0.117 | 1.36 | | (0.93, 2.00) |
| *SREBF/RAI1* | rs11868035 | 0.996 | 0.16/0.14 | A | | | | 0.548 | 0.572 | 0.90 | (0.63, 1.28) | | 0.132 | 0.47 | | (0.18, 1.26) |
| *COQ7* | rs11343 | 0.991 | 0.01/0.01 | T | | | | 0.944 | 0.944 | 0.95 | (0.24, 3.83) | | 0.944 | 0.95 | | (0.24, 3.85) |
| *FGF20* | rs591323 | 0.177 | 0.43/0.42 | A | | | | 0.705 | 0.716 | 1.05 | (0.81, 1.36) | | 0.520 | 0.88 | | (0.61, 1.29) |
| *GALC* | rs8005172 | 0.925 | 0.36/0.28 | T | | | | 0.009 | 0.010 | 0.69 | (0.53, 0.92) | | 0.015 | 2.17 | | (1.18, 4.15) |
| *NMD3* | rs34016896 | 0.736 | 0.41/0.45 | T | | | | 0.170 | 0.174 | 1.20 | (0.93, 1.54) | | 0.777 | 1.07 | | (0.68, 1.69) |
| *ELOVL7* | rs2694528 | 0.994 | 0.07/0.07 | C | | | | 0.723 | 0.721 | 0.92 | (0.56, 1.49) | | 0.714 | 0.91 | | (0.54, 1.52) |
| *FAM171A1* | rs10906923 | 0.972 | 0.46/0.48 | C | | | | 0.592 | 0.593 | 1.07 | (0.83, 1.38) | | 0.402 | 1.20 | | (0.78, 1.84) |
| *ZNF184* | rs9468199 | 0.814 | 0.25/0.18 | A | | | | 0.008 | 0.008 | 1.52 | (1.12, 2.08) | | 0.057 | 1.43 | | (0.99, 2.07) |
| *MMP16* | rs60298754 | 0.997 | 0.06/0.07 | T | | | | 0.459 | 0.458 | 0.83 | (0.50, 1.37) | | 0.376 | 0.79 | | (0.46, 1.34) |
| *SATB1* | rs4073221 | 0.967 | 0.05/0.03 | G | | | | 0.078 | 0.079 | 1.80 | (0.93, 3.49) | | 0.100 | 1.77 | | (0.91, 3.58) |
| *ALAS1, TLR9, DNAH1, BAP1, PHF7, NISCH, STAB1, ITIH3, ITIH4* | rs143918452 | 0.933 | 0.02/0.01 | G | | | | 0.128 | 0.125 | 0.42 | (0.13, 1.33) | | - | - | | - |
| *NCKIPSD,CDC71* | rs12497850 | 0.993 | 0.06/0.05 | G | | | | 0.502 | 0.507 | 1.21 | (0.69, 2.13) | | 0.478 | 1.24 | | (0.69, 2.25) |
| *ITPKB* | rs4653767 | 0.999 | 0.26/0.29 | C | | | | 0.383 | 0.382 | 0.88 | (0.67, 1.17) | | 0.405 | 0.86 | | (0.60, 1.23) |
| *IL1R2* | rs34043159 | 0.965 | 0.47/0.54 | C | | | | 0.034 | 0.036 | 1.31 | (1.02, 1.69) | | 0.101 | 1.41 | | (0.94, 2.12) |
| *ATP6V0A1,PSMC3IP,*  *TUBG2* | rs601999 | 0.768 | 0.03/0.04 | C | | | | 0.620 | 0.624 | 1.19 | (0.60, 2.36) | | 0.980 | - | | - |
| *USP25* | rs2823357 | 0.995 | 0.27/0.29 | A | | | | 0.444 | 0.458 | 0.90 | (0.68, 1.19) | | 0.814 | 0.96 | | (0.67, 1.37) |
| *SH3GL2* | rs13294100 | <.0001 | 0.48/0.52 | T | | | | - | - | - | - | | - | - | | - |
| Candidate Gene | SNP | Genetic Power | Dominant Model (adjusted)^b^ | | | | | | Recessive Model | | | | Recessive Model (adjusted)^b^ | | | |
|  |  |  | *p* | OR | | 95%CI | | | *p* | OR | 95%CI | | *p* | OR | | 95%CI |
| *TOX3* | rs4784227 | 0.050 | 0.721 | 0.94 | | (0.65, 1.35) | | | 0.507 | 0.76 | (0.33, 1.69) | | 0.605 | 0.81 | | (0.35, 1.81) |
| *SORBS3, PDLIM2, C8orf58, BIN3* | rs2280104 | 0.164 | 0.100 | 1.38 | | (0.94, 2.03) | | | 0.526 | 0.73 | (0.26, 1.93) | | 0.515 | 0.72 | | (0.26, 1.91) |
| *SREBF/RAI1* | rs11868035 | 0.091 | 0.132 | 0.47 | | (0.16, 1.21) | | | 0.990 | 1.00 | (0.67, 1.50) | | 0.966 | 1.01 | | (0.67, 1.52) |
| *COQ7* | rs11343 | 0.052 | 0.924 | 0.93 | | (0.22, 4.01) | | | - | - | - | | - | - | | - |
| *FGF20* | rs591323 | 0.057 | 0.562 | 0.89 | | (0.61, 1.30) | | | 0.154 | 0.72 | (0.46, 1.13) | | 0.146 | 0.71 | | (0.45, 1.12) |
| *GALC* | rs8005172 | 0.069 | 0.020 | 2.11 | | (1.14, 4.07) | | | 0.056 | 1.42 | (0.99, 2.04) | | 0.036 | 1.47 | | (1.03, 2.12) |
| *NMD3* | rs34016896 | 0.111 | 0.724 | 1.09 | | (0.68, 1.73) | | | 0.072 | 0.71 | (0.48, 1.03) | | 0.060 | 0.69 | | (0.47, 1.01) |
| *ELOVL7* | rs2694528 | 0.072 | 0.879 | 0.96 | | (0.57, 1.62) | | | 0.970 | 1.06 | (0.04, 26.78) | | 0.940 | 1.11 | | (0.04, 28.37) |
| *FAM171A1* | rs10906923 | 0.066 | 0.427 | 1.19 | | (0.77, 1.83) | | | 0.949 | 0.99 | (0.66, 1.47) | | 0.920 | 0.98 | | (0.66, 1.46) |
| *ZNF184* | rs9468199 | 0.072 | 0.063 | 1.42 | | (0.98, 2.07) | | | 0.007 | 0.22 | (0.06, 0.60) | | 0.005 | 0.20 | | (0.06, 0.56) |
| *MMP16* | rs60298754 | 0.183 | 0.296 | 0.75 | | (0.43, 1.28) | | | 0.970 | 1.06 | (0.04, 26.79) | | 0.995 | 0.99 | | (0.04, 25.29) |
| *SATB1* | rs4073221 | 0.489 | 0.144 | 1.67 | | (0.85, 3.42) | | | 0.980 | - | - | | 0.980 | - | | - |
| *ALAS1, TLR9, DNAH1, BAP1, PHF7, NISCH, STAB1, ITIH3, ITIH4* | rs143918452 | 0.958 | - | - | | - | | | 0.137 | 2.44 | (0.80, 8.98) | | 0.131 | 2.47 | | (0.81, 9.12) |
| *NCKIPSD,CDC71* | rs12497850 | 0.184 | 0.398 | 1.29 | | (0.72, 2.35) | | | 0.972 | 1.05 | (0.04, 26.68) | | 0.987 | 0.98 | | (0.04, 24.90) |
| *ITPKB* | rs4653767 | 0.102 | 0.368 | 0.85 | | (0.59, 1.21) | | | 0.608 | 1.19 | (0.60, 2.38) | | 0.425 | 1.33 | | (0.66, 2.73) |
| *IL1R2* | rs34043159 | 0.088 | 0.111 | 1.40 | | (0.93, 2.11) | | | 0.074 | 0.69 | (0.45, 1.04) | | 0.076 | 0.69 | | (0.45, 1.04) |
| *ATP6V0A1,PSMC3IP,*  *TUBG2* | rs601999 | 0.150 | 0.980 | - | | - | | | 0.492 | 0.78 | (0.38, 1.58) | | 0.530 | 0.80 | | (0.39, 1.62) |
| *USP25* | rs2823357 | 0.092 | 0.812 | 0.96 | | (0.67, 1.37) | | | 0.200 | 1.51 | (0.81, 2.89) | | 0.167 | 1.58 | | (0.83, 3.05) |
| *SH3GL2* | rs13294100 | - | - | - | | - | | | - | - | - | | - | - | | - |
| Candidate Gene | SNP | Overdominant model | | | | | Overdominant model (adjusted)^b^ | | | | | Number of sample tested | | | | |
|  |  | *p* | OR | | 95%CI | | *p* | | OR | 95%CI | | Case | | | Control | |
| *TOX3* | rs4784227 | 0.589 | 1.11 | | (0.76, 1.61) | | 0.544 | | 1.12 | (0.77, 1.64) | | 248 | | | 235 | |
| *SORBS3, PDLIM2, C8orf58, BIN3* | rs2280104 | 0.174 | 0.76 | | (0.51, 1.13) | | 0.152 | | 0.74 | (0.50, 1.11) | | 248 | | | 236 | |
| *SREBF/RAI1* | rs11868035 | 0.482 | 1.17 | | (0.76, 1.79) | | 0.501 | | 1.16 | (0.75, 1.78) | | 248 | | | 237 | |
| *COQ7* | rs11343 | 0.944 | 1.05 | | (0.25, 4.49) | | 0.924 | | 1.07 | (0.25, 4.60) | | 248 | | | 236 | |
| *FGF20* | rs591323 | 0.077 | 1.38 | | (0.97, 1.98) | | 0.084 | | 1.38 | (0.95, 1.98) | | 248 | | | 236 | |
| *GALC* | rs8005172 | 0.690 | 0.93 | | (0.65, 1.33) | | 0.502 | | 0.88 | (0.61, 1.27) | | 248 | | | 235 | |
| *NMD3* | rs34016896 | 0.139 | 1.31 | | (0.92, 1.88) | | 0.132 | | 1.32 | (0.92, 1.89) | | 248 | | | 235 | |
| *ELOVL7* | rs2694528 | 0.716 | 1.10 | | (0.65, 1.86) | | 0.889 | | 1.04 | (0.61, 1.76) | | 250 | | | 237 | |
| *FAM171A1* | rs10906923 | 0.520 | 0.89 | | (0.62, 1.27) | | 0.565 | | 0.90 | (0.63, 1.29) | | 248 | | | 236 | |
| *ZNF184* | rs9468199 | 0.503 | 0.88 | | (0.60, 1.28) | | 0.574 | | 0.90 | (0.61, 1.31) | | 248 | | | 236 | |
| *MMP16* | rs60298754 | 0.439 | 1.24 | | (0.72, 2.14) | | 0.345 | | 1.31 | (0.75, 2.28) | | 248 | | | 235 | |
| *SATB1* | rs4073221 | 0.133 | 0.59 | | (0.29, 1.16) | | 0.188 | | 0.63 | (0.31, 1.24) | | 248 | | | 235 | |
| *ALAS1, TLR9, DNAH1, BAP1, PHF7, NISCH, STAB1, ITIH3, ITIH4* | rs143918452 | 0.137 | 0.41 | | (0.11, 1.25) | | 0.131 | | 0.40 | (0.11, 1.23) | | 240 | | | 234 | |
| *NCKIPSD,CDC71* | rs12497850 | 0.465 | 0.80 | | (0.43, 1.45) | | 0.392 | | 0.77 | (0.42, 1.40) | | 248 | | | 236 | |
| *ITPKB* | rs4653767 | 0.566 | 1.11 | | (0.77, 1.60) | | 0.618 | | 1.10 | (0.76, 1.58) | | 250 | | | 237 | |
| *IL1R2* | rs34043159 | 0.901 | 1.02 | | (0.72, 1.46) | | 0.879 | | 1.03 | (0.72, 1.47) | | 249 | | | 238 | |
| *ATP6V0A1,PSMC3IP,*  *TUBG2* | rs601999 | 0.382 | 1.38 | | (0.67, 2.89) | | 0.418 | | 1.35 | (0.66, 2.83) | | 248 | | | 236 | |
| *USP25* | rs2823357 | 0.607 | 0.91 | | (0.63, 1.31) | | 0.569 | | 0.90 | (0.62, 1..30) | | 248 | | | 236 | |
| *SH3GL2* | rs13294100 | - | - | | - | | - | | - | - | | 238 | | | 240 | |
| CI= confidence interval; HWE = Hardy-Weinberg Equilibrium; MAF = minor allele frequency; OR = odds ratio; PD = Parkinson’s Disease; SNP = single nucleotide polymorphism.  ^a^*p* value, OR and 95% CI were obtained from risk analysis and refer to the risk allele.  ^b^ Adjusted for age and gender.  ^c^ *p* value was calculated by Cochran-Armitage trend test for additive model. | | | | | | | | | | | | | | | | |

Table S3 Association of SNPs of candidate genes and odds ratio to LOPD risk

| Candidate Gene | SNP | HWE *p* value | MAF (case/control) | Allele | | | | | | | | | Dominant Model | | | |
| --- | --- | --- | --- | --- | --- | --- | --- | --- | --- | --- | --- | --- | --- | --- | --- | --- |
|  |  |  |  | Effect allele | | | | *p^a^* | *p*_trend_^c^ | OR^a^ | 95%CI^a^ | | *p* | OR | | 95%CI |
| *TOX3* | rs4784227 | 0.999 | 0.25/0.23 | T | | | | 0.497 | 0.493 | 1.11 | (0.82, 1.51) | | 0.660 | 1.09 | | (0.75, 1.58) |
| *SORBS3, PDLIM2, C8orf58, BIN3* | rs2280104 | 0.972 | 0.21/0.16 | T | | | | 0.062 | 0.066 | 1.38 | (0.98, 1.94) | | 0.073 | 1.44 | | (0.97, 2.13) |
| *SREBF/RAI1* | rs11868035 | 0.179 | 0.16/0.14 | A | | | | 0.538 | 0.560 | 0.89 | (0.62, 1.28) | | 0.157 | 0.48 | | (0.16, 1.29) |
| *COQ7* | rs11343 | 1.000 | 0.01/0.01 | T | | | | 0.895 | 0.894 | 1.10 | (0.27, 4.42) | | 0.894 | 1.10 | | (0.26, 4.70) |
| *FGF20* | rs591323 | 0.295 | 0.43/0.42 | A | | | | 0.645 | 0.656 | 1.06 | (0.81, 1.39) | | 0.670 | 0.92 | | (0.62, 1.35) |
| *GALC* | rs8005172 | 0.838 | 0.36/0.28 | T | | | | 0.016 | 0.016 | 0.71 | (0.53, 0.94) | | 0.014 | 2.22 | | (1.19, 4.29) |
| *NMD3* | rs34016896 | 0.886 | 0.40/0.45 | T | | | | 0.104 | 0.105 | 1.25 | (0.96, 1.62) | | 0.507 | 1.18 | | (0.73, 1.92) |
| *ELOVL7* | rs2694528 | 1.000 | 0.06/0.07 | C | | | | 0.258 | 0.258 | 0.73 | (0.43, 1.26) | | 0.230 | 0.71 | | (0.40, 1.24) |
| *FAM171A1* | rs10906923 | 0.993 | 0.46/0.48 | C | | | | 0.539 | 0.542 | 1.09 | (0.84, 1.41) | | 0.414 | 1.20 | | (0.77, 1.88) |
| *ZNF184* | rs9468199 | 0.999 | 0.23/0.18 | A | | | | 0.062 | 0.056 | 1.36 | (0.98, 1.89) | | 0.150 | 1.33 | | (0.90, 1.95) |
| *MMP16* | rs60298754 | 0.940 | 0.06/0.07 | T | | | | 0.476 | 0.468 | 0.82 | (0.49, 1.40) | | 0.470 | 0.82 | | (0.47, 1.41) |
| *SATB1* | rs4073221 | 1.000 | 0.05/0.03 | G | | | | 0.073 | 0.076 | 1.84 | (0.93, 3.62) | | 0.099 | 1.80 | | (0.90, 3.69) |
| *ALAS1, TLR9, DNAH1, BAP1, PHF7, NISCH, STAB1, ITIH3, ITIH4* | rs143918452 | 0.953 | 0.02/0.01 | G | | | | 0.117 | 0.115 | 0.40 | (0.12, 1.31) | | - | - | | - |
| *NCKIPSD,CDC71* | rs12497850 | 0.938 | 0.06/0.05 | G | | | | 0.633 | 0.631 | 1.15 | (0.64, 2.08) | | 0.519 | 1.22 | | (0.66, 2.26) |
| *ITPKB* | rs4653767 | 0.967 | 0.26/0.29 | C | | | | 0.414 | 0.415 | 0.89 | (0.66, 1.19) | | 0.370 | 0.84 | | (0.58, 1.22) |
| *IL1R2* | rs34043159 | 0.936 | 0.47/0.54 | C | | | | 0.056 | 0.059 | 1.29 | (0.99, 1.67) | | 0.158 | 1.36 | | (0.89, 2.07) |
| *ATP6V0A1,PSMC3IP,*  *TUBG2* | rs601999 | 0.681 | 0.03/0.04 | C | | | | 0.515 | 0.522 | 1.27 | (0.61, 2.63) | | 0.980 | - | | - |
| *USP25* | rs2823357 | 0.959 | 0.26/0.29 | A | | | | 0.395 | 0.412 | 0.88 | (0.66, 1.18) | | 0.683 | 0.93 | | (0.64, 1.34) |
| *SH3GL2* | rs13294100 | <0.001 | 0.50/0.52 | T | | | | - | - | - | - | | - | - | | - |
| Candidate Gene | SNP | Genetic Power | Dominant Model (adjusted)^b^ | | | | | | Recessive Model | | | | Recessive Model (adjusted)^b^ | | | |
|  |  |  | *p* | OR | | 95%CI | | | *p* | OR | 95%CI | | *p* | OR | | 95%CI |
| *TOX3* | rs4784227 | 0.085 | 0.897 | 1.03 | | (0.70, 1.51) | | | 0.399 | 0.71 | (0.31, 1.59) | | 0.521 | 0.76 | | (0.32, 1.76) |
| *SORBS3, PDLIM2, C8orf58, BIN3* | rs2280104 | 0.145 | 0.067 | 1.46 | | (0.97, 2.21) | | | 0.983 | - | - | | 0.982 | - | | - |
| *SREBF/RAI1* | rs11868035 | 0.095 | 0.381 | 0.63 | | (0.21, 1.73) | | | 0.933 | 1.02 | (0.67, 1.55) | | 0.967 | 0.99 | | (0.64, 1.53) |
| *COQ7* | rs11343 | 0.060 | 0.832 | 0.86 | | (0.19, 3.76) | | | - | - | - | | - | - | | - |
| *FGF20* | rs591323 | 0.060 | 0.803 | 0.95 | | (0.64, 1.42) | | | 0.188 | 0.73 | (0.46, 1.16) | | 0.179 | 0.72 | | (0.44, 1.16) |
| *GALC* | rs8005172 | 0.068 | 0.011 | 2.39 | | (1.23, 4.81) | | | 0.096 | 1.37 | (0.95, 1.99) | | 0.046 | 1.48 | | (1.01, 2.18) |
| *NMD3* | rs34016896 | 0.118 | 0.505 | 1.19 | | (0.72, 1.97) | | | 0.062 | 0.69 | (0.46, 1.02) | | 0.062 | 0.68 | | (0.45, 1.02) |
| *ELOVL7* | rs2694528 | 0.288 | 0.479 | 0.81 | | (0.45, 1.44) | | | 0.950 | 0.92 | (0.04, 23.24) | | 0.773 | 1.51 | | (0.06, 38.95) |
| *FAM171A1* | rs10906923 | 0.074 | 0.505 | 1.17 | | (0.74, 1.85) | | | 0.838 | 0.96 | (0.64, 1.44) | | 0.834 | 0.96 | | (0.63, 1.46) |
| *ZNF184* | rs9468199 | 0.136 | 0.242 | 1.23 | | (0.85, 1.89) | | | 0.054 | 0.32 | (0.09, 0.96) | | 0.066 | 0.33 | | (0.09, 1.00) |
| *MMP16* | rs60298754 | 0.181 | 0.517 | 0.83 | | (0.46, 1.46) | | | 0.980 | - | - | | 0.980 | - | | - |
| *SATB1* | rs4073221 | 0.478 | 0.106 | 1.82 | | (0.89, 3.82) | | | 0.980 | - | - | | 0.980 | - | | - |
| *ALAS1, TLR9, DNAH1, BAP1, PHF7, NISCH, STAB1, ITIH3, ITIH4* | rs143918452 | 0.722 | - | - | | - | | | 0.127 | 2.53 | (0.81, 9.45) | | 0.151 | 2.44 | | (0.76, 9.31) |
| *NCKIPSD,CDC71* | rs12497850 | 0.108 | 0.264 | 1.43 | | (0.76, 2.72) | | | 0.980 | - | - | | 0.980 | - | | - |
| *ITPKB* | rs4653767 | 0.093 | 0.466 | 0.87 | | (0.59, 1.27) | | | 0.797 | 1.09 | (0.55, 2.21) | | 0.670 | 1.17 | | (0.57, 2.48) |
| *IL1R2* | rs34043159 | 0.102 | 0.217 | 1.31 | | (0.85, 2.04) | | | 0.093 | 0.69 | (0.45, 1.06) | | 0.084 | 0.68 | | (0.44, 1.06) |
| *ATP6V0A1,PSMC3IP,*  *TUBG2* | rs601999 | 0.242 | 0.981 | - | | - | | | 0.386 | 0.72 | (0.33, 1.51) | | 0.399 | 0.72 | | (0.32, 1.54) |
| *USP25* | rs2823357 | 0.100 | 0.634 | 0.91 | | (0.62, 1.34) | | | 0.247 | 1.47 | (0.77, 2.89) | | 0.195 | 1.57 | | (0.80, 3.19) |
| *SH3GL2* | rs13294100 | - | - | - | | - | | | - | - | - | | - | - | | - |
| Candidate Gene | SNP | Overdominant model | | | | | Overdominant model (adjusted)^b^ | | | | | Number of sample tested | | | | |
|  |  | *p* | OR | | 95%CI | | *p* | | OR | 95%CI | | Case | | | Control | |
| *TOX3* | rs4784227 | 0.960 | 0.99 | | (0.68, 1.45) | | 0.864 | | 1.04 | (0.70, 1.54) | | 215 | | | 235 | |
| *SORBS3, PDLIM2, C8orf58, BIN3* | rs2280104 | 0.144 | 0.74 | | (0.49, 1.1) | | 0.125 | | 0.72 | (0.47, 1.10) | | 215 | | | 236 | |
| *SREBF/RAI1* | rs11868035 | 0.571 | 1.14 | | (0.73, 1.77) | | 0.655 | | 1.11 | (0.71, 1.76) | | 215 | | | 237 | |
| *COQ7* | rs11343 | 0.894 | 0.91 | | (0.21, 3.89) | | 0.832 | | 1.17 | (0.27, 5.14) | | 215 | | | 236 | |
| *FGF20* | rs591323 | 0.144 | 1.32 | | (0.91, 1.92) | | 0.190 | | 1.29 | (0.88, 1.90) | | 215 | | | 236 | |
| *GALC* | rs8005172 | 0.880 | 0.97 | | (0.67, 1.41) | | 0.636 | | 0.91 | (0.62, 1.34) | | 215 | | | 235 | |
| *NMD3* | rs34016896 | 0.211 | 1.27 | | (0.87, 1.84) | | 0.208 | | 1.28 | (0.87, 1.88) | | 215 | | | 235 | |
| *ELOVL7* | rs2694528 | 0.219 | 1.43 | | (0.81, 2.58) | | 0.508 | | 1.22 | (0.68, 2.22) | | 217 | | | 237 | |
| *FAM171A1* | rs10906923 | 0.618 | 0.91 | | (0.63, 1.32) | | 0.711 | | 0.93 | (0.63, 1.36) | | 215 | | | 236 | |
| *ZNF184* | rs9468199 | 0.480 | 0.87 | | (0.59, 1.28) | | 0.644 | | 0.91 | (0.61, 1.36) | | 215 | | | 236 | |
| *MMP16* | rs60298754 | 0.630 | 1.15 | | (0.66, 2.01) | | 0.684 | | 1.13 | (0.63, 2.03) | | 215 | | | 235 | |
| *SATB1* | rs4073221 | 0.135 | 0.59 | | (0.28, 1.17) | | 0.148 | | 0.58 | (0.28, 1.20) | | 215 | | | 235 | |
| *ALAS1, TLR9, DNAH1, BAP1, PHF7, NISCH, STAB1, ITIH3, ITIH4* | rs143918452 | 0.127 | 0.40 | | (0.11, 1.23) | | 0.151 | | 0.41 | (0.11, 1.31) | | 217 | | | 234 | |
| *NCKIPSD,CDC71* | rs12497850 | 0.424 | 0.78 | | (0.42, 1.44) | | 0.195 | | 0.65 | (0.34, 1.24) | | 215 | | | 236 | |
| *ITPKB* | rs4653767 | 0.438 | 1.16 | | (0.80, 1.69) | | 0.607 | | 1.11 | (0.75, 1.64) | | 217 | | | 237 | |
| *IL1R2* | rs34043159 | 0.829 | 1.04 | | (0.72, 1.51) | | 0.678 | | 1.08 | (0.74, 1.59) | | 216 | | | 238 | |
| *ATP6V0A1,PSMC3IP,*  *TUBG2* | rs601999 | 0.281 | 1.53 | | (0.72, 3.42) | | 0.316 | | 1.50 | (0.69, 3.40) | | 215 | | | 236 | |
| *USP25* | rs2823357 | 0.786 | 0.95 | | (0.65, 1.39) | | 0.779 | | 0.94 | (0.64, 1.40) | | 215 | | | 236 | |
| *SH3GL2* | rs13294100 | - | - | | - | | - | | - | - | | 206 | | | 240 | |
| CI= confidence interval; HWE = Hardy-Weinberg Equilibrium; LOPD = late onset Parkinson’s Disease; MAF = minor allele frequency; OR = odds ratio; PD = Parkinson’s Disease; SNP = single nucleotide polymorphism.  ^a^*p* value, OR and 95% CI were obtained from risk analysis and refer to the risk allele.  ^b^ Adjusted for age and gender.  ^c^ *p* value was calculated by Cochran-Armitage trend test for additive model. | | | | | | | | | | | | | | | | |

Table S4 Association of SNPs of candidate genes and odds ratio to EOPD risk

| Candidate Gene | SNP | HWE *p* value | MAF (case/control) | Allele | | | | | | | | Dominant Model | | | |
| --- | --- | --- | --- | --- | --- | --- | --- | --- | --- | --- | --- | --- | --- | --- | --- |
|  |  |  |  | Effect allele | | | *p^a^* | *p*_trend_^c^ | OR^a^ | 95%CI^a^ | | *p* | OR | | 95%CI |
| *TOX3* | rs4784227 | 0.501 | 0.12/0.23 | T | | | 0.038 | 0.036 | 0.45 | (0.21, 0.97) | | 0.026 | 0.37 | | (0.14,0.84) |
| *SORBS3, PDLIM2, C8orf58, BIN3* | rs2280104 | 0.805 | 0.14/0.16 | T | | | 0.637 | 0.639 | 0..84 | (0.40, 1.76) | | 0.854 | 0.93 | | (0.39, 2.03) |
| *SREBF/RAI1* | rs11868035 | 0.231 | 0.15/0.14 | A | | | 0.862 | 0.866 | 0.94 | (0.46, 1.93) | | 0.278 | 0.40 | | (0.09, 2.83) |
| *FGF20* | rs591323 | 0.249 | 0.41/0.42 | A | | | 0.898 | 0.900 | 0.97 | (0.57, 1.63) | | 0.338 | 0.70 | | (0.33, 1.48) |
| *GALC* | rs8005172 | 0.904 | 0.36/0.28 | T | | | 0.102 | 0.092 | 0.64 | (0.37, 1.10) | | 0.284 | 1.89 | | (0.51, 5.58) |
| *NMD3* | rs34016896 | 0.531 | 0.40/0.45 | T | | | 0.751 | 0.750 | 0.92 | (0.55, 1.54) | | 0.279 | 0.63 | | (0.28, 1.52) |
| *ELOVL7* | rs2694528 | 0.832 | 0.06/0.07 | C | | | 0.032 | 0.029 | 2.24 | (1.05, 4.77) | | 0.024 | 2.60 | | (1.10, 5.82) |
| *FAM171A1* | rs10906923 | 0.885 | 0.46/0.48 | C | | | 0.952 | 0.953 | 0.98 | (0.59, 1.65) | | 0.710 | 1.18 | | (0.51, 3.08) |
| *ZNF184* | rs9468199 | 0.294 | 0.23/0.18 | A | | | 0.0001 | 0.0001 | 2.82 | (1.62, 4.88) | | 0.024 | 2.34 | | (1.12, 4.95) |
| *MMP16* | rs60298754 | 0.185 | 0.06/0.07 | T | | | 0.927 | 0.733 | 0.83 | (0.28, 2.41) | | 0.411 | 0.59 | | (0.14, 1.79) |
| *SATB1* | rs4073221 | 1.000 | 0.05/0.03 | G | | | 0.760 | 0.489 | 1.55 | (0.43, 5.55) | | 0.493 | 1.58 | | (0.35, 5.19) |
| *ALAS1, TLR9, DNAH1, BAP1, PHF7, NISCH, STAB1, ITIH3, ITIH4* | rs143918452 | 1.000 | 0.02/0.01 | G | | | 0.479 | 0.589 | 0.55 | (0.06, 5.00) | | - | - | | - |
| *NCKIPSD,CDC71* | rs12497850 | 0.217 | 0.06/0.05 | G | | | 0.529 | 0.376 | 1.60 | (0.59, 4.36) | | 0.611 | 1.34 | | (0.37, 3.81) |
| *ITPKB* | rs4653767 | 0.690 | 0.26/0.29 | C | | | 0.620 | 0.615 | 0.86 | (0.48, 1.55) | | 0.924 | 0.97 | | (0.46, 2.01) |
| *IL1R2* | rs34043159 | 0.970 | 0.47/0.54 | C | | | 0.142 | 0.144 | 1.47 | (0.88, 2.47) | | 0.199 | 1.84 | | (0.77, 5.10) |
| *ATP6V0A1,PSMC3IP,*  *TUBG2* | rs601999 | 0.964 | 0.03/0.04 | C | | | 0.774 | 0.769 | 0.83 | (0.24, 2.91) | | - | - | | - |
| *USP25* | rs2823357 | 0.848 | 0.26/0.29 | A | | | 0.997 | 0.997 | 1.00 | (0.57, 1.76) | | 0.630 | 1.20 | | (0.58, 2.50) |
| *SH3GL2* | rs13294100 | <0.001 | 0.50/0.52 | T | | | - | - | - | - | | - | - | | - |
| Candidate Gene | SNP | Genetic Power | Dominant Model (adjusted)^b^ | | | | | Recessive Model | | | | Recessive Model (adjusted)^b^ | | | |
|  |  |  | *p* | OR | 95%CI | | | *p* | OR | 95%CI | | *p* | OR | | 95%CI |
| *TOX3* | rs4784227 | 0.036 | 0.235 | 0.50 | (0.15, 1.50) | | | 0.670 | 1.57 | (0.29, 29.20) | | 0.956 | 3.59 | | (0.09, 36.36) |
| *SORBS3, PDLIM2, C8orf58, BIN3* | rs2280104 | 0.078 | 0.364 | 0.58 | (0.17, 1.78) | | | 0.987 | - | - | | 0.992 | - | | - |
| *SREBF/RAI1* | rs11868035 | 0.055 | 0.158 | 0.16 | (0.01, 2.55) | | | 0.814 | 0.90 | (0.36, 2.03) | | 0.562 | 1.40 | | (0.43, 4.31) |
| *FGF20* | rs591323 | 0.052 | 0.384 | 0.64 | (0.23, 1.78) | | | 0.341 | 0.66 | (0.29, 1.65) | | 0.495 | 0.66 | | (0.21, 2.28) |
| *GALC* | rs8005172 | 0.063 | 0.857 | 0.85 | (0.12, 4.47) | | | 0.128 | 1.80 | (0.86, 3.92) | | 0.954 | 1.03 | | (0.37, 2.86) |
| *NMD3* | rs34016896 | 0.058 | 0.160 | 0.45 | (0.15, 1.41) | | | 0.641 | 0.83 | (0.39, 1.87) | | 0.608 | 0.76 | | (0.26, 2.26) |
| *ELOVL7* | rs2694528 | 0.256 | 0.335 | 1.75 | (0.53, 5.41) | | | 0.989 | - | - | | 0.994 | - | | - |
| *FAM171A1* | rs10906923 | 0.052 | 0.642 | 0.76 | (0.24, 2.53) | | | 0.654 | 1.21 | (0.54, 3.00) | | 0.528 | 1.45 | | (0.48, 4.87) |
| *ZNF184* | rs9468199 | 0.180 | 0.340 | 1.62 | (0.59, 4.38) | | | <0.0001 | 0.06 | (0.02, 0.23) | | 0.014 | 0.12 | | (0.02, 0.62) |
| *MMP16* | rs60298754 | 0.074 | 0.335 | 0.43 | (0.06, 2.04) | | | 0.163 | 0.14 | (0.01, 3.51) | | 0.187 | 0.12 | | (0.00, 3.86) |
| *SATB1* | rs4073221 | 0.122 | 0.381 | 2.17 | (0.33, 11.11) | | | - | - | - | | - | - | | - |
| *ALAS1, TLR9, DNAH1, BAP1, PHF7, NISCH, STAB1, ITIH3, ITIH4* | rs143918452 | 0.332 | - | - | - | | | 0.595 | 1.83 | (0.09, 12.84) | | 0.281 | 5.19 | | (0.17, 81.12) |
| *NCKIPSD,CDC71* | rs12497850 | 0.224 | 0.903 | 0.91 | (0.17, 3.85) | | | 0.162 | 0.14 | (0.01, 3.50) | | 0.290 | 0.10 | | (0.00, 5.91) |
| *ITPKB* | rs4653767 | 0.071 | 0.822 | 0.89 | (0.32, 2.41) | | | 0.326 | 2.79 | (0.55, 50.96) | | 0.860 | 1.23 | | (0.18, 25.73) |
| *IL1R2* | rs34043159 | 0.154 | 0.740 | 1.22 | (0.39, 4.30) | | | 0.281 | 0.64 | (0.29, 1.49) | | 0.369 | 0.60 | | (0.20, 1.88) |
| *ATP6V0A1,PSMC3IP,*  *TUBG2* | rs601999 | 0.081 | - | - | - | | | 0.769 | 1.21 | (0.27, 3.85) | | 0.328 | 2.11 | | (0.40, 8.78) |
| *USP25* | rs2823357 | 0.050 | 0.921 | 1.05 | (0.38, 2.89) | | | 0.424 | 1.84 | (0.51, 11.76) | | 0.432 | 2.08 | | (0.40, 17.32) |
| *SH3GL2* | rs13294100 | - | - | - | - | | | - | - | - | | - | - | | - |
| Candidate Gene | SNP | Overdominant model | | | | Overdominant model (adjusted)^b^ | | | | | Number of sample tested | | | | |
|  |  | *p* | OR | 95%CI | | *p* | | OR | 95%CI | | Case | | | Control | |
| *TOX3* | rs4784227 | 0.035 | 2.69 | (1.14, 7.45) | | 0.233 | | 1.98 | (0.66, 7.27) | | 33 | | | 235 | |
| *SORBS3, PDLIM2, C8orf58, BIN3* | rs2280104 | 0.861 | 0.93 | (0.42, 2.21) | | 0.447 | | 1.58 | (0.51, 5.55) | | 33 | | | 236 | |
| *SREBF/RAI1* | rs11868035 | 0.487 | 1.39 | (0.58, 3.88) | | 0.969 | | 0.98 | (0.30, 3.54) | | 33 | | | 237 | |
| *FGF20* | rs591323 | 0.101 | 1.90 | (0.90, 4.23) | | 0.166 | | 2.07 | (0.75, 6.06) | | 33 | | | 236 | |
| *GALC* | rs8005172 | 0.333 | 0.70 | (0.33, 1.45) | | 0.874 | | 0.92 | (0.34, 2.53) | | 33 | | | 235 | |
| *NMD3* | rs34016896 | 0.196 | 1.63 | (0.78, 3.51) | | 0.106 | | 2.33 | (0.86, 6.82) | | 33 | | | 235 | |
| *ELOVL7* | rs2694528 | 0.019 | 0.37 | (0.17, 0.88) | | 0.332 | | 0.57 | (0.18, 1.86) | | 33 | | | 237 | |
| *FAM171A1* | rs10906923 | 0.474 | 0.77 | (0.36, 1.59) | | 0.862 | | 0.92 | (0.34, 2.49) | | 33 | | | 236 | |
| *ZNF184* | rs9468199 | 0.897 | 0.95 | (0.45, 2.13) | | 0.563 | | 1.38 | (0.48, 4.28) | | 33 | | | 236 | |
| *MMP16* | rs60298754 | 0.236 | 2.44 | (0.69, 15.54) | | 0.121 | | 5.03 | (0.81, 50.63) | | 33 | | | 235 | |
| *SATB1* | rs4073221 | 0.493 | 0.63 | (0.19, 2.86) | | 0.381 | | 0.46 | (0.09, 3.04) | | 33 | | | 235 | |
| *ALAS1, TLR9, DNAH1, BAP1, PHF7, NISCH, STAB1, ITIH3, ITIH4* | rs143918452 | 0.595 | 0.55 | (0.08, 10.88) | | 0.281 | | 0.19 | (0.01, 6.03) | | 33 | | | 234 | |
| *NCKIPSD,CDC71* | rs12497850 | 0.971 | 0.98 | (0.31, 4.31) | | 0.558 | | 1.70 | (0.34, 12.50) | | 33 | | | 236 | |
| *ITPKB* | rs4653767 | 0.655 | 0.85 | (0.41, 1.78) | | 0.883 | | 1.08 | (0.40, 3.07) | | 33 | | | 237 | |
| *IL1R2* | rs34043159 | 0.800 | 0.91 | (0.44, 1.89) | | 0.623 | | 1.28 | (0.48, 3.55) | | 33 | | | 238 | |
| *ATP6V0A1,PSMC3IP,*  *TUBG2* | rs601999 | 0.769 | 0.83 | (0.26, 3.67) | | 0.328 | | 0.47 | (0.11, 2.47) | | 33 | | | 236 | |
| *USP25* | rs2823357 | 0.319 | 0.69 | (0.33, 1.45) | | 0.548 | | 0.74 | (0.27, 2.03) | | 33 | | | 236 | |
| *SH3GL2* | rs13294100 | - | - | - | | - | | - | - | | 32 | | | 240 | |
| CI= confidence interval; EOPD = Early Onset Parkinson’s Disease; HWE = Hardy-Weinberg Equilibrium; MAF = minor allele frequency; OR = odds ratio; PD = Parkinson’s Disease; SNP = single nucleotide polymorphism.  ^a^*p* value, OR and 95% CI were obtained from risk analysis and refer to the risk allele.  ^b^ Adjusted for age and gender.  ^c^ *p* value was calculated by Cochran-Armitage trend test for additive model. | | | | | | | | | | | | | | | |

Table S5 Accelerated failure time model of genetic models of single nucleotide polymorphisms

|  |  | Models of SNPs | *p* value | Coefficients Value | 95% CI | Loglik results of models |
| --- | --- | --- | --- | --- | --- | --- |
| PD vs. Control | Model 1 | Dominant model of rs8005172 | 4.29 × 10^-2^ | -3.37 | (-6.62, -0.11) | -1028.2 |
|  | Model 2 | Recessive model of rs8005172 | 7.93 × 10^-2^ | -1.89 | (-4.00, 0.22) | -1028.7 |
|  | Model 3 | Recessive model of rs9468199 | 1.44 × 10^-3^ | 8.15 | (3.14, 13.17) | -1026.9 |
|  | Model 4 | Dominant model of rs8005172 | 3.40 × 10^-2^ | -3.49 | (-6.72, -0.26) | -1023.2 |
|  |  | Recessive model of rs9468199 | 1.18 × 10^-3^ | 8.23 | (3.25, 13.20) |  |
|  | Model 5 | Recessive model of rs8005172 | 1.22 × 10^-1^ | -1.67 | (-3.77, 0.45) | -1024.2 |
|  |  | Recessive model of rs9468199 | 2.34 × 10^-3^ | 7.77 | (2.76, 12.76) |  |
| LOPD vs. Control | Model 1 | Dominant model of rs8005172 | 2.85 × 10^-2^ | -2.98 | (-5.65, -0.31) | -822.9 |
|  | Model 2 | Recessive model of rs8005172 | 2.29 × 10^-1^ | -1.08 | (-2.84, 0.68) | -824.5 |
| EOPD vs. Control | Model 1 | Dominant model of rs2694528 | 1.73 × 10^-2^ | -17.44 | (-31.80, -3.08) | -224.5 |
|  | Model 2 | Dominant model of rs9468199 | 2.62 × 10^-2^ | -14.80 | (-27.85, -1.75) | -224.6 |
|  | Model 3 | Recessive model of rs9468199 | 1.19 × 10^-5^ | 40.77 | (22.53, 59.02) | -218 |
|  | Model 4 | Overdominant model of rs2694528 | 1.31 × 10^-2^ | 18.2 | (3.83, 32.57) | -224.3 |
|  | Model 5 | Dominant model of rs2694528 | 1.89 × 10^-2^ | -17.02 | (-31.23, -2.81) | -221.4 |
|  |  | Dominant model of rs9468199 | 2.29 × 10^-2^ | -14.78 | (-27.51, -2.05) |  |
|  | Model 6 | Dominant model of rs2694528 | 6.72 × 10^-3^ | -18.25 | (-31.45, -5.05) | -214.1 |
|  |  | Recessive model of rs9468199 | 4.94 × 10^-6^ | 41.73 | (23.82, 59.64) |  |
|  | Model 7 | Overdominant model of rs2694528 | 1.25 × 10^-2^ | 18.11 | (3.90, 32.31) | -221.1 |
|  |  | Dominant model of rs9468199 | 2.04 × 10^-2^ | -15.01 | (-27.69, -2.32) |  |
|  | Model 8 | Overdominant model of rs2694528 | 5.04 × 10^-3^ | 18.89 | (5.69, 32.09) | -213.9 |
|  |  | Recessive model of rs9468199 | 4.84 × 10^-6^ | 41.61 | (23.77, 59.45) |  |
| CI: confidence interval; EOPD: Early Onset Parkinson’s Disease; LOPD: Late Onset Parkinson’s Disease; PD: Parkinson’s Disease; SNP: single nucleotide polymorphisms; | | | | | | |
